# Molecular epidemiology study of *Mycobacterium tuberculosis* and its susceptibility to anti-tuberculosis drugs in Indonesia

**DOI:** 10.1186/s12879-015-1101-y

**Published:** 2015-08-22

**Authors:** Vivi Lisdawati, Nelly Puspandari, Lutfah Rif’ati, Triyani Soekarno, Melatiwati M, Syamsidar K, Lies Ratnasari, Nur Izzatun, Ida Parwati

**Affiliations:** Institute for Vector and Reservoir Disease Control Research and Development (IVRDCRD), Salatiga, Indonesia; Center for Biomedical and Basic Technology of Health (CBBTH), NIHRD, MoH-RI, Jakarta, Indonesia; Department of Clinical Pathology, Dr. Hasan Sadikin Hospital, Bandung, Indonesia

## Abstract

**Background:**

Genotyping of *Mycobacterium tuberculosis* helps to understand the molecular epidemiology of tuberculosis and to address evolutionary questions about the disease spread. Certain genotypes also have implications for the spread of infection and treatment. Indonesia is a very diverse country with a population with multiple ethnicities and cultures and a history of many trade and tourism routes. This study describes the first attempt to map the molecular epidemiology of TB in the Indonesian archipelago.

**Method:**

From 2008 to 2011, 404 clinical specimens from sputum-smear (SS+) TB patients, age ≥15 years, were collected from 16 TB referral primary health centers (PHC) in 16 provincial capitals in Indonesia. Susceptibility testing to first line drugs was conducted for 262 samples using the agar proportion method as per WHO guidelines. Spoligotyping was done on all samples.

**Results:**

Ninety-three of the 404 samples (23 %) were from the Beijing family, making it the predominant family in the country. However, the geographic distribution of the family varied by region with 86/294 (29.3 %) in the western region, 6/72 (8.3 %) in the central region, and 2/72 (2.8 %) in the eastern region (*p* < 0.001). The predominant genotype in the central and eastern regions was from the East-African-Indian (EAI) family, comprising 15.3 % (11/72), and 26.3 % (10/38) of the isolates, respectively. Drug susceptibility to first-line anti-TB drugs was tested in 262 isolates. 162 (61.8 %) isolates were susceptible to all TB drugs, 70 (26.7 %) were mono-resistant 16 (6.1 %) were poly-resistant, and 14 (5.4 %) were multi-drug resistant (MDR). The proportion of Beijing family isolates in the susceptible, mono-resistant, poly-resistant, and MDR groups was 33/162 (20.4 %), 28/70 (40.0 %), 6/16 (37.5 %), and 3/14 (21.4 %), respectively. Overall, resistance of the Beijing family isolates to any of the first line TB drugs was significantly higher than non-Beijing families [37/71 (52.1 %) vs. 63/191 (33.0 %) (*p*-value = 0.003)].

**Conclusion:**

The distribution of *Mycobacterium tuberculosis* genotypes in Indonesia showed high genetic diversity and tended to vary by geographic regions. Drug susceptibility testing confirmed that the Beijing family of *M.tb* in Indonesia exhibited greater resistance to first line anti-TB drugs than did other families.

## Background

Tuberculosis (TB) remains a major global health problem and ranks as the second leading cause of death from an infectious disease worldwide. There were 9 million new TB cases and 1.5 million TB related deaths in 2013. Among these deaths, it was estimated that more than 200,000 were due to MDR-TB, a high proportion of the 480,000 incident cases of MDR-TB [1]. A serious problem has been the emergence of extensively-drug resistance TB (XDR-TB) comprising 9 % of the MDR cases. The treatment of MDR- and XDR-TB is substantially more costly and difficult to treat, with higher rates of treatment failure and mortality, as compared to drug-susceptible tuberculosis [2, 3].

Indonesia is considered a high-burden country for TB, ranked fifth in TB incidence (460,000 new TB patients each year) worldwide [1]. An Indonesian National Basic Health Survey in 2010 reported 289 cases/100,000 population [4]. According to the Indonesian TB prevalence survey in 2004, differences in TB prevalence among different regions was observed. For example, 82 cases/100,000 population were reported in the Java-Bali region, compared with 343 cases/100,000 population in Eastern Indonesia and 217 cases/100,000 population in the Sumatra-Kalimantan region [5]. The differences in the incidences is likely multifactorial and may include such factors such as income level, hygiene, education and availability of health care facilities [2, 4, 6].

In addition to the high prevalence of TB cases, drug-resistant DR-TB is also an important problem in Indonesia. WHO estimated 1.9 % of new TB cases and 12 % of previously treated TB cases in Indonesia have MDR-TB [1]. Several studies reveal that MDR-TB occurs more frequently in patients with a previous history of pulmonary TB treatment, particularly those with inadequate treatments (including monotherapy, insufficient duration of therapy or inconsistency of adherence), and in patients with co-morbidities, including human immunodeficiency virus (HIV) infection or diabetes mellitus (DM) [3, 4]. There are risk factors for the occurrence of mutations in the specific genes of the bacteria that may lead to drug-resistance. The prevalence of MDR-TB in Indonesia also varies geographically. According to the TB resistance survey conducted between 2004 and 2007, the prevalence of MDR-TB in Central Java, Makassar and Papua was 1.9 %, 4.1 % and 2.0 %, respectively [2]. Therefore, it is important to understand both the geographic distribution of bacterial genotypes as well as the susceptibility of individuals within different regions according to the possibility of genetic susceptibility mutations [7].

Although MDR-TB is emerging throughout Indonesia, there is only one study of nearly 900 patients that evaluates drug resistance and M. tb genotypes. This study did not find drug resistance associated with certain genotypes. However, it did find that the Beijing genotype, found more frequently in West Java (Bandung) than in West Timor (Kupang), was associated with treatment failure [8, 9].

Here we describe the initial mapping of 404 *M. tb* bacterial genotypes using spoligotyping, the sensitivity of *M. tb* isolates to first line anti-TB drugs, and the association between the genotypes and drug sensitivity, which will complement the previous publication of this study [10].

## Methods

**Fig. 1:**
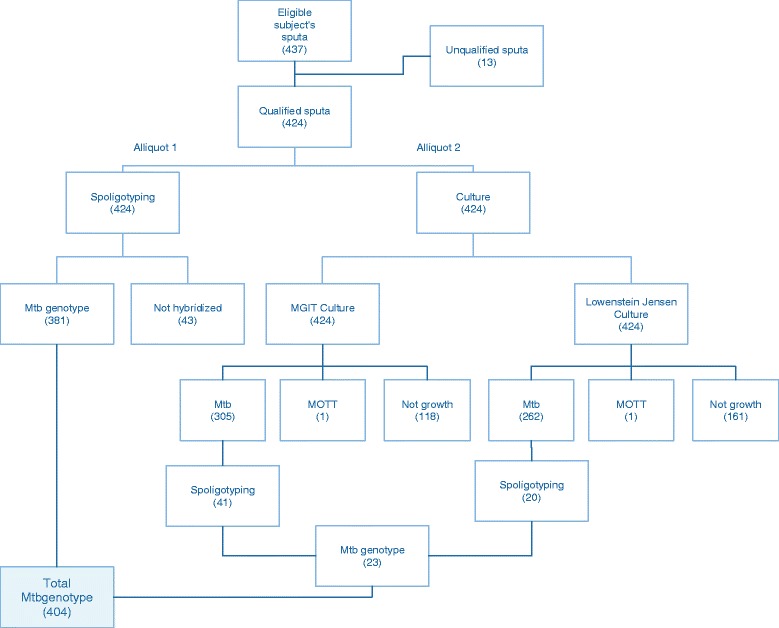
The scheme of the laboratory procedures conducted to sample sputa from 16 TB refferal health center in 16 provincial capitals in Indonesia

A cross-sectional study was conducted in 16 TB referral primary health centers (PHC) that had the ability to perform microscopic sputum examination. The study was conducted in 16 provincial capitals in Indonesia, including five provincial capitals in Sumatra (Bandar Lampung, Palembang, Padang, Medan and Pekan Baru), four in Java (Serang, Jakarta, Bandung and Surabaya) two in Kalimantan (Banjarmasin and Pontianak), two in Sulawesi (Makassar and Menado) and three in Eastern Indonesia (Mataram, Ambon and Sorong) between 2008 and 2010. Ethical clearance was obtained from the Ethics Review Committee of NIHRD of Indonesia. Laboratory procedures were performed according to the algorithm in Fig. 1.

### Subjects and samples

Individual sputum samples were collected from 437 subjects who met the inclusion criteria: i) age ≥ 15 years, ii) newly diagnosed TB or in treatment less than two months, iii) sputum smear positive, and iv) willingness to sign informed consent. Subjects whose sputa were not available were not included in the study. Sputum samples collected by PHC technicians from suspected TB patients included a spot sputum on day 1 and an early morning sputum and a spot sputum on day 2. All patients were trained by the PHC technicians how to produce sputum as per national TB guidelines to ensure that all sputum from different PHC were comparable. All sputum were sent to and processed by the National Institute of Health Research and Development (NIHRD) in Jakarta. NIHRD’s laboratory technicians then chose one of the three sputa based on the quality of the sputum (macroscopically thick, pus-like, sometimes bloody, and microscopically having more leukocytes than epithelial cells). Sputum samples from 13 subjects were rejected due to contamination during specimen processing.

Laboratory Procedures:

A. Sputum decontamination
  Sputum specimens were decontaminated using MycoPrep™, a solution containing sodium hydroxide and N-acetyl-L-cysteine, in Falcon tubes, following sputum decontamination guidelines [11], in Biosafety cabinet class II type A2 using Personal Protective Equipment (PPE) for Biosafety Laboratory Level-3 (BSL-3). This suspension was divided into two aliquots and stored in an incubator at 37 °C. for further process . One aliquot was prepared for DNA extraction (for genotyping and spoligotyping) and the second aliquot was prepared for culture (for genotyping and susceptibility testing). Spoligotyping and genotyping test were performed first on sputum to shorten the time to obtain results.

B. DNA Extraction, Liquid Culture Media BACTEC MGIT-960 and Solid Culture Media Lowenstein Jensen (LJ)
  The first aliquot above was used for genotyping. *M. tb* DNA was isolated according to the method of DNA extraction, as described in the kit manual (®Qiagen) using the manufacturers protocol. The extracted DNA was eluted from QIAspin columns in a final volume of 200 μL AE buffer and stored at −70°C until used for genotyping. When the genotype pattern could not be obtained from DNA isolated from the first aliquot, due to the limitation of direct sputum processing, genotyping was then attempted from the second aliquot.
  The second aliquot was divided into two. The first part was added to the Falcon tubes that contained BACTEC™ MGIT™ Oleic acid, Albumin, Dextrose, Catalase (OADC) growth supplement and MGIT PANTA antimicrobic mixture (®BD. Location exact details of kit). *M. tb* liquid MGIT Bactec culture was performed as per the kit manual prepared by the manufacturer. The second part was added to the Lowenstein-Jensen (L-J)media for solid culture as per WHO guidelines [12]. All preparation was done in biosafety cabinet Class II Type A2 using PPE for BSL-3. When the Bactec MGIT-960 media culture showed *M. tb* growth at 2 – 4 weeks, some isolates were used for genotyping. When the L-J media culture showed *M. tb* growth at 2 – 8 weeks, the isolates were used for first line anti-TB susceptibility test and some isolates were also used for genotyping.

C. Spoligotyping
  Testing was performed on DNA isolates from the first aliquot and from some cultures according to the manufacturing procedure for spoligotyping for *M.tb’s* DNA [13], using primer DRA: 5'-GCT TTT GGG TCT GAC GAC-3 'labeled with biotin at the 5' end and primer DRB: 5'-CCG AGA GGG GAC GGA AAC-3 ', milliQ as negative control and *M. tb* H37Rv and M.bovis BCG as positive control, to avoid false negative or false positive results. To minimize false negative and false positive results, we separated the PCR mix chamber with sample preparation room and implemented appropriate techniques during mini-blotter use to avoid air-bubles. Amplification of DNA was conducted using a thermocycler (AB System). PCR products were then hybridized using the mini-blotter MN45 equipment as per instructions from the manufacturer. Hybridization results were compared with the data base patterns on Spoligotipe Data Base (SPOLDB4/SITVIT) and the dendrogram was constructed using software BioNumerics v6.0.

D. First line anti-TB Drug Susceptibility Test
  Susceptibility testing was conducted in accordance with the agar proportion method by WHO [12]. First line drugs used in this test were Streptomycin, Isoniazid, Rifampicin and Ethambutol (SIRE) with susceptibility cutoff concentrations [4; 0.2; 40; 2 (μg/ml)]. Results were interpreted by observing the presence or absence of *M. tb* bacteria on 28 day cultured samples compared to negative control. *M. tb* strains were considered resistant when isolates showed at least 1 % resistance by counting the bacteria growth as per the WHO formula and were considered sensitive when <1 % isolates showed resistance. When day 28 results were resistant, the reading was not repeated and strains were considered resistant. However, when day 28 reading resulted in sensitive results, samples were re-read on day 42. Readings on day 42 were considered definitive readings.

## Results

A total of 437 subjects were consented, with the majority from the western region (302/69.1 %), followed by the eastern (76/17.4 %) and central regions (59/13.5 %). The western, central and eastern regions consisted of 11, 2 and 3 PHCs, respectively. Each PHC contributed 20 to 30 subjects. The subjects were 15–74 years of age, with a median of 32 years. The ratio between males and females was 1.8:1. This ratio was found in 12 PHC, except in Makassar where it was equal, and in Serang, Banjarmasin and Sorong where females were slightly more than males. Details of the regions and cities where the PHCs are located are listed in Table 1.

**Table 1:** The list of the cities, number of screened subjects and results of spoligotyping and resistance testing

Cities	Screened	Spoligotyping	L-J cultured	Resistance						Susceptible (%)
		(%)		Str	INH	Rif	Eth	MDR	Poly-drug	
								INH+ RIF	Resistance	
Western Region	302	284 (186/98)	201	17	20	3	12	12	13	124 (62)
Padang	29	25 (17/8)	18	2	2	0	0	0	2	12 (66.7)
Pekan Baru	30	29 (23/6)	14	2	1	0	1	1	2	7 (50)
Palembang	30	30 (21/9)	16	0	2	0	1	0	2	11 (68.8)
Lampung	28	26 (18/8)	20	2	2	1	2	5	0	8 (40)
Medan	20	19 (14/5)	16	2	2	2	1	0	0	9 (56.3)
Serang	29	29 (14/15)	24	2	2	0	1	0	1	18 (75)
DKI Jakarta	28	28 (19/9)	16	2	0	0	0	3	1	10 (62.5)
Bandung	26	24 (16/8)	22	1	4	0	3	1	4	9 (40.9)
Surabaya	30	30 (17/13)	16	2	1	0	0	0	0	13 (81.3)
Banjarmasin	25	17 (8/9)	22	2	2	0	2	0	0	16 (72.7)
Pontianak	27	27 (19/8)	17	0	2	0	1	2	1	11 (64.7)
Central Region	59	59 (36/23)	28	2	6	0	0	2	2	16 (57.1)
Makassar	30	30 (15/15)	18	2	3	0	0	1	2	10 (55.6)
Manado	29	29 (21/8)	10	0	3	0	0	1	0	6 (60)
Eastern Region	76	61 (36/25)	33	7	1	1	1	0	1	22 (66.7)
Mataram	28	13 (12/1)	9	1	1	0	0	0	0	7 (77.8)
Ambon	28	28 (15/13)	15	2	0	1	1	0	1	10 (66.7)
Sorong	20	20 (9/11)	9	4	0	0	0	0	0	5 (55.6)
Total	437	404 (258/146)	262	26	27	4	13	14	16	162 (61.8)

Thirteen of the 437 sputa from these patients were contaminated during specimen processing, and were excluded from further analysis. Spoligotyping and culture growth (in liquid MGIT Bactec 960 media and solid L-J media) were performed on 424 sputa. During spoligotyping 381 sputa were hybridized and 43 did not hybridize. Culture on liquid and solid media resulted in growth of 305 and 262 *M. tb* samples, respectively. Spoligotyping repeated using the isolates from these culture, resulting in 23 additional hybridizations of 43 subjects who had not hybridized to spoligotyping performed from the direct sputa specimens. In total, the genotypes of *M. tb* from 404 subject could be characterized, both from sputa and culture in liquid and solid media (Fig. 1).

Based on spoligotyping results, molecular epidemiology data demonstrated genotype diversity of *M. tb* in Indonesia [Table 2], where 404 patterns of *M. tb* strains match with 149 patterns on the spoligotype database (SPOLDB4/SITVIT). Overall our study found that the largest number of *M. tb* isolates in Indonesia belonged to the Beijing family (93/404, 23.0 %), followed by East African Indian/EAI-type (Family F) (83/404, 20.5 %) and Latin American-Mediterranean/LAM-type (Family D) (50/404, 12.4 %). Fifty-seven patterns of *M. tb* were not in the SPOLDB4 database and were designated as orphan type (14.1 %). The Beijing family was the most predominant (86/29.3 %) genotype in the western region, followed by the EA family (62/21.1 %) genotype, LAM family (10.9 %) genotype and orphan (10.5 %) group. In contrast, Beijing family was only found in 6 *M. tb* (8.3 %) isolates in the central region and in 1 *M. tb* (2.6 %) isolate in eastern region. The difference in prevalence of the Beijing family in the western region compared to the other regions was significant (p < 0.01). The EA (11/15.3 %) and LAM genotypes (10/13.9 %) was predominant in the central region. The EA was also the most predominant in the eastern region (10/26.3 %), followed by LAM and T genotypes (each 8/21.1 %) (Table 2).

**Table 2:** Details of *M. tb* spoligotyping results divided into each region in Indonesia

Genotype*Mtb*	Western region freq (%)	Central region freq (%)	Eastern region freq (%)	Total
Beijing strain (fam. I)	86 (29.3)	6 (8.3)	1(2.6)	93
H genotype (fam. A)	23 (7.8)	9 (12.5)	2 (5.3)	34
LAM genotype (fam. D)	32 (10.9)	10 (13.9)	8 (21.1)	50
EA genotype (fam. F)	62 (21.1)	11 (15.3)	10 (26.3)	83
T genotype (fam. C)	20 (6.8)	4 (5.6)	8 (21.1)	32
U genotype (fam. G and H)	21 (6.8)	9 (12.5)	2 (5.3)	32
MANU1 genotype (fam. others)	6 (2)	-	-	6
MANU2 genotype (fam. others)	3 (1)	2 (2.8)	-	5
X1 genotype(fam. B)	6 (2)	-	-	6
CAS genotype (fam. others)	3 (1)	-	-	3
PINI genotype (fam. others)	-	-	1 (2.6)	1
Africanum genotype (fam. others)	-	1 (1.4)	-	1
Orphan	31 (10.5)	20 (27.8)	6 (15.8)	57
H37Rv	1 (0.3)	-	-	1
TOTAL	294	72	38	404

Drug susceptibility results from 262 culture positive samples were matched with their respective spoligotyping results. Details of drug susceptibility results to first line drugs, MDR and poly-resistant, non MDR in each town, region and across all sites are detailed in Table 1. The proportion of *M. tb* isolates that were susceptible to all first line TB drugs in Indonesia was 61.8 % and this was not different between the regions (western: 62 %, central: 57.1 %, eastern: 66.7 %). There were some cities where the prevalence of drug sensitive TB was higher such as Surabaya (81.3 %), Mataram (77.8 %) and Banjarmasin (72.7 %). In contrast, in Lampung, Bandung, and Pakan Baru, the prevalence of drug sensitive TB was ≤ 50 %.

The proportion of MDR across all sites was 5.3 %. The majority of this was concentrated in the western and central regions (6 % and 5.3 %). Interestingly, no MDR strains were found in 33 *M. tb* isolates from the eastern part of Indonesia. In western Indonesia, MDR *M. tb* isolates were identified, in order of frequency, from Lampung (5), Jakarta (3), Pontianak (2), Pekan Baru (1), Bandung (1), and in central Indonesia from Makassar (1) and Manado (1). Of note, although almost 60 % (13/22) of *M. tb* isolates from Bandung had some resistance to first line TB drugs, only one was MDR. Resistance to single drugs was most frequent (36.3 %), followed by poly-drug, non-MDR (18.2 %) and MDR (4.5 %).

Analysis of *M. tb* susceptibility in the Beijing family and non-Beijing family groups is described in Table 3. The Beijing family demonstrated the highest mono-resistance [28/70(40.0 %)], followed by poly-resistant, drug sensitive TB, and MDR groups [6/16(37.5 %)], [33/162(20.4 %)] and [3/14(21.4 %)], respectively.

**Table 3:** The susceptibility and resistance of Beijing and Non-Beijing strain groups to first line anti-TB drugs

Susceptibility/Resistance	*M.tuberculosis* genotype		Total
	Beijing	Non-Beijing	
Susceptible	33	129	162
Monoresistance	28	42	70
Streptomycin	6	20	26
INH	11	16	27
Rifampicin	3	1	4
Ethambutol	8	5	13
Multi Drug Resistance (MDR)	3	11	14
Poly-resistance	6	10	16
SI	2	5	7
SR	0	0	0
SE	0	1	1
IE	4	2	6
RE	0	0	0
SIE	0	1	1
SER	0	1	1
Total	70	192	262

The resistance amongst the Beijing family to any first line TB drug was significantly higher than in the non-Beijing families (52.9 %% vs 32.8 %, *p* < 0.01). This higher resistance was largely due to the mono-resistant group (40 % vs 21.9 %, *p* < 0.01), and with some contribution from the poly-resistant group (8.6 % vs 5.2 %, *p* = 0.31). This was not seen when comparison was made between MDR strains in each group (4.3 % vs 5.7 %, *p* = 0.64). Further analysis of the resistance to each first line drug, individually or in combination with other drugs, revealed that the prevalence of resistance to INH was higher in the Beijing family (28.6 %) compared to (18.2 %) in non-Beijing family but this was not statistically significant. Similarly, the prevalence of resistance to rifampicin in Beijing family (8.6 %) was not significantly higher than non-Beijing family (6.8 %). Resistance to ethambutol was significantly higher in the Beijing family compared to non-Beijing groups (17.1 % vs 5.2 % *p* < 0.01). In contrast, resistance to streptomycin in the Beijing group was not significantly lower than in the non-Beijing family (11.4 % vs 14.6 %).

## Discussion

This study adds to the data available in Indonesia with respect to *M. tb* starins and drug resistance in the various regions of the country. The majority of subjects in this study are between 15–54 years, reflecting the age group with high mobility as a routine activity in this age group as known. The proportion of males in our study was higher than female which could suggest that TB prevalence was higher in men than in women. This is consistent with the results of National Indonesian Basic Health Research in 2010, [4] which showed that the prevalence of TB in Indonesia is higher in men. An epidemiological study in India in 1986–2002 also found gender bias in TB patients [14]. Research to determine the role of paternal relationship or Y chromosome on the suseptibility of Mtb, along with research on genetic influences sensitivity exposed by the host with a particular type Mtb is underway [15, 16]. Recent study showed that the specific gene like CYP7A1 gene, which codes for cholesterol 7a-hydroxylase, an enzyme involved in cholesterol catabolism, may play a role in susceptibility to TB in human population [7]. However, given the strong patrilineal cultural factors in Indonesian society, where men are considered more important in all aspects, including in access to medical treatment should be considered.

This study used spoligotyping to obtain genotyping data. This method has proven to be an effective and efficient method for the study of genotypes in the population because it can be used to construct a dendrogram structure that describes the circulation of the group (clade) of *M. tb*, while also being able to recognize the existing lines [16, 17]. When compared with other fingerprinting methods such as restriction fragment length polymorphism (RFLP) with a probe of the sequence IS6110, spoligotyping has an advantage because this method has a probe on the membrane which in addition to distinguishing genotype can simultaneously recognize strains of *M. tb* complex, which can not be done by probes on conventional RFLP method in a single reaction process [8, 9]. Due to limits in the number of primer used in standard spoligotyping, spoligotyping generally equipped with Mycobacterial interspersed repetitive unit-variable number of tandem repeat (MIRU-VNTR) typing method that has the ability to provide more information as it uses more loci [18, 19].

Some samples showed no hybridization on membrane spoligotyping. Some references indicate that the spoligotyping often fails to produce hybridization patterns from DNA samples derived directly from sputum specimens, though with appropriate modifications of extraction techniques this can be improved [9]. Hybridization failure may be associated with small numbers of bacteria present in the sample or the possibility that the bacteria are dead so that there can be no replication. This is evident from the results of the evaluation, in which some samples which did not show a pattern of hybridization also failed to show growth of colonies on culture media.

This study produced several important findings. The Beijing family genotype was the predominant *M. tb* family in West Indonesian region (Java, Sumatra, Kalimantan), but was the minority in Eastern Indonesia. Based on data of *M. tb* distribution that the W-Beijing strain family is associated with the migration of ethnic Chinese [20–22], we speculate that this may be the reason for the dominance of this family in the main trading cities in Sumatra, Java and Kalimantan. As is well known, ethnic Chinese first came to Indonesia by sea for trade and the sampling sites in the western and central regions were port cities in ancient times. Similar results have been reported in Parwati, et al. [8].

In contrast, in the region with inaccessible migration paths, such as the eastern region, the W-Beijing strain family may not have arrived and other isolates predominate, such as the East African-Indian (EAI) and Haarlem (H)-like in Madagascar [23], the type of Latin American-Mediterranean (LAM) also observed in Turkey [24] or the typical type of Central Asian Strain (CAS)-like in Pakistan [25]. Considering the migration theory, this may also illustrate that the movement of *M. tb* strains in east region in Indonesia also follows the movement of the human population of Polynesian and African regions as mentioned earlier.

Our study also found that the proportion of resistance to anti-TB drugs within Beijing strains family was significantly higher than non-Beijing strains family. This is consistent with previous research suggesting that a number of Beijing family have putative mutator genes and cause changes in DNA repair that will drive the direction of resistance mutations [26, 27]. Research on Beijing strains from Germany and Russia showed mutations in a high proportion of the genes rpoB S531L and embB306. A study in Hong Kong reports the association between Beijing strains with phenotypic resistance to INH]. [28, 22] These mutations indicate any possibility of mutations that can arise in bacteria gene, especially M. tb gene. Our study also found no MDR case in the Eastern region. However, as a study in Northern Malawi showed, for all isolates of the Beijing still susceptible to anti TB drugs [29], further study needs to be conducted.

We also found several important *M. tb* types such as the H type. Recent molecular studies found this type should be closely monitored because the 3R gene (DNA repair, recombination, and replication) reveal a high tendency to mutate, similar to the Beijing strains, even though the mechanism is not clear [9]. The prevalence of MDR-TB was relatively high and the orphan group was the most common isolate found in the central region. However, the limitation of spoligotyping as a genotyping method has also to be considered when finding too many orphan *M. tb* types.

Genotype studies on population using spoligotyping such as in this study is beneficial to provide the dendogram structure that could clarify *M. tb* clade circulation, together with identifying prevalent strains [30]. Dendrogram data would be useful for comparing patterns between countries. This comparison will determine which countries share similar dendogram patterns. These countries may then share their experience in TB surveillance programs.

This study has several limitations. First, the difficulty to maintain the cold chain sample may influence the results of bacterial culture. In addition, genotyping analysis was only conducted by spoligotyping without combining it with another method, such as MIRU – VNTR typing, which would provide more definitive differentiation of strains of each family of *M. tb* because MIRU typing has more loci to complete the genotyping results. A third limitation of the present study is the relatively small sample size used for susceptibility testing that may not represent the true pattern of resistance for the population.

## Conclusion

In summary, spoligotyping on 404 sputum specimens from TB patients highlights the diversity of *M. tb* genotypes in Indonesia. The majority of *M. tb* strains from Java, Sumatra and Kalimantan was family I (type W-Beijing family strains) whereas eastern Indonesia was dominated by family F (type EAI) and family D (type LAM) isolates. Resistance to anti-TB drugs was more common in W-Beijing family strains, which, in turn, were more commonly found in Java. Hence, this finding is an important consideration in the management of TB in regions with high rates of resistance and suggests that there is a need for the government to enhance the TB surveillance program in Indonesia.
